# Development of Café-Au-Lait-Macule Lesions in a Patient After Dupilumab Therapy for Atopic Dermatitis

**DOI:** 10.7759/cureus.42581

**Published:** 2023-07-27

**Authors:** Afnan Hasanain, Abdulellah I Aleissa, Wasan AlQurashi

**Affiliations:** 1 Dermatology, King Faisal Specialist Hospital and Research Centre, Jeddah, SAU; 2 Medical School, King Abdulaziz University Faculty of Medicine, Jeddah, SAU; 3 Dermatology, King Abdulaziz University Hospital, Jeddah, SAU

**Keywords:** dermatology, café-au-lait macules, dupilumab dermatologic reaction, dupilumab, atopic dermatitis

## Abstract

Atopic dermatitis (AD) is a chronic immune-mediated disease characterized by intense pruritis, causing inflammation, itching, and redness of the skin. Dupilumab is a human monoclonal antibody that has been approved for the treatment of atopic dermatitis. It has also been linked with various adverse effects, most of them confined to the injection site. Café-au-lait-macules are benign pigmented lesions of the skin, usually seen in people with genetic disorders. We present a case of café-au-lait macules as an adverse effect of dupilumab therapy in a patient with atopic dermatitis. The patient in this case had been receiving dupilumab therapy for atopic dermatitis. The eczematous lesions had seen improvement; however, the patient presented with CALMs on follow-up, which seem to be linked with dupilumab therapy.

## Introduction

Cafe-au-lait macules (CALMs) are well-defined, pigmented macules or patches that range in color from light to dark brown [1]. They are caused by an increase in the number of melanocytes, leading to increased production of melanin in the epidermis and the formation of café-au-lait (CAL) spots [2]. They are associated more commonly with genetic conditions such as neurofibromatosis type 1 (NF 1), among others [3]. Atopic dermatitis (AD) or eczema is a chronic condition that results in skin irritation, redness, and inflammation. It is a widespread condition that typically manifests in childhood, but anyone can contract the illness at any time [4]. Dupilumab is a drug that has recently been approved for the treatment of atopic dermatitis. It is a human monoclonal antibody that targets the signaling mechanisms of the cytokines interleukin-4 and interleukin-13 [5]. We present the case of a patient who developed CALMs after subcutaneous (SC) administration of Dupixent (dupilumab) 300mg for atopic dermatitis.

## Case presentation

A 25-year-old patient presented to the outpatient department with the complaint of erythematous round lesions on the hand and face for a month. She had a history of atopic dermatitis. She also reported a family history of lupus.

**Figure 1:**
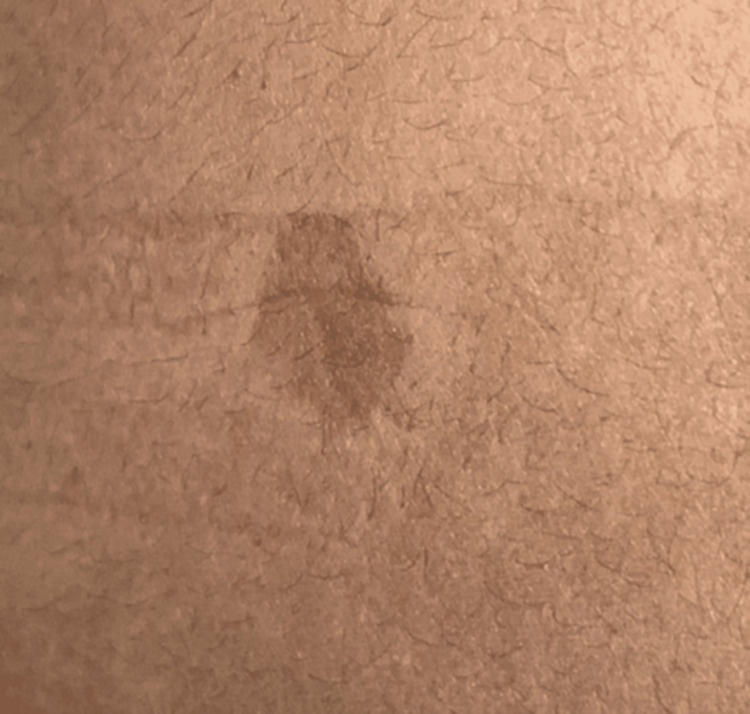
Hyperpigmented macules and patches over the lower limb

She had been receiving dupilumab 300mg SC every two weeks, tacrolimus, and mometasone. She had also been receiving levothyroxine 50 mcg therapy for hypothyroidism. Atopic dermatitis was seen to be improved with dupilumab, after which she reported eczematous lesions and did not use any topical therapy for three weeks. On examination, hyperpigmented macules and patches were seen over the lower limb (LL) (Figure 1) and the back. She also had eczematous lesions over the right lower eyelid and the right thumb.

A working diagnosis of café-au-lait macules with atopic dermatitis was made at this point. The treatment plan of Protopic twice a day, continuing on Dupixent, a moisturizing cream, and vitamin A and D ointment, was made while giving instructions for follow-up to the clinic in six months.

## Discussion

The patient in this case had developed café-au-lait macules after receiving dupilumab therapy for atopic dermatitis. Dupilumab is a human monoclonal antibody targeted against interleukin-4 and interleukin-13. The blockade by dupilumab of these key drivers of type 2 helper T-cell (Th2)-mediated inflammation could help in the treatment of related diseases, including atopic dermatitis [6]. Dupilumab was approved by the U.S. Food and Drug Administration (FDA) in 2017 for the treatment of adult patients with moderate-to-severe atopic dermatitis whose disease is not adequately controlled with topical prescription therapies or when those therapies are not advisable [7].

The adverse effects reported with dupilumab therapy include dupilumab-induced lichen planus [8], in which the patient developed lichenoid lesions involving the buccal walls, trunk, and extremities after 11 months of drug administration. Another case of a patient developing pustular psoriasis after dupilumab therapy has been reported [9]. Dupilumab therapy has been associated with several skin-related adverse effects, including but not limited to keratosis pilaris and rosacea. These adverse events have been attributed to the association of dupilumab with the downregulation of T-helper 2 cell activation by blocking the interleukin-4/ interleukin-13 pathway, leading to a TH1/TH2 imbalance. This imbalance may cause a shift towards a TH1-mediated immune response, causing the associated drug-induced conditions mentioned above. As we see in this case, there is an appearance of café-au-lait macules after three years of dupilumab therapy [1].

"Café-au-lait macules" (CALMs), which are flat skin lesions characterized by hyperpigmentation, commonly manifest either at birth (congenitally) or during early infancy. With age, they might multiply and enlarge in size. They can be found on any part of the body and range in color from light brown to dark brown, but the trunk and extremities are where they are most frequently found. The French phrase "café au lait" translates to "coffee with milk" [1].

CALMs, or café-au-lait macules, can be classified into two main types. The first type, known as the "coast of California," is characterized by regular and well-defined margins. These CALMs can appear as single or multiple spots and vary in size from a few millimeters to several centimeters (>20cm). On the other hand, the second type, referred to as the "coast of Maine," is less common and typically larger and solitary, with an irregular margin. The "coast of Maine" pattern is associated with a segmental pigmentary disorder, whereas the "coast of California" pattern is observed in neurofibromatosis 1 (NF1) and related conditions [1].

In this case, the dupilumab therapy for the patient was not discontinued, and the patient was given the treatment plan of dupilumab for atopic dermatitis since discontinuing it might lead to worsening the condition. To date, the patient is responding well to the therapy. For further assessment of macules, the patient is advised to follow up at the clinic after six months.

## Conclusions

This case highlights the incidence of CALM-like lesions in patients reported after dupilumab therapy for atopic dermatitis. Dupilumab is in phase 3 clinical trials and has been approved by the FDA. This case shows a possible adverse effect of dupilumab and provides a direction for healthcare providers to keep the adverse effects of dupilumab therapy in mind while planning a treatment strategy for their patients.

## References

[1] Jha SK, Mendez MD (2023). Cafe au lait macules. StatPearls.

[2] (2023). Café-Au-Lait Spots. https://my.clevelandclinic.org/health/diseases/22627-cafe-au-lait-spots.

[3] Anderson S (2020). Café au lait macules and associated genetic syndromes. J Pediatr Health Care.

[4] (2023). National Institute of Arthritis and Musculoskeletal and Skin Diseases. Atopic dermatitis: treatment, symptoms & causes. https://www.niams.nih.gov/health-topics/atopic-dermatitis.

[5] Tameez Ud Din A, Malik I, Arshad D, Tameez Ud Din A (2020). Dupilumab for atopic dermatitis: the silver bullet we have been searching for?. Cureus.

[6] Beck LA, Thaçi D, Hamilton JD (2014). Dupilumab treatment in adults with moderate-to-severe atopic dermatitis. N Engl J Med.

[7] Simpson EL, Bieber T, Guttman-Yassky E (2016). Two phase 3 trials of dupilumab versus placebo in atopic dermatitis. N Engl J Med.

[8] Kern L, Kleinheinrich L, Feldmann R, Sator P, Stella A, Breier F (2022). Dupilumab-induced lichen planus: a case with oral and cutaneous eruptions. Case Rep Dermatol.

[9] Jia X, Li C, Wu J, Liu Q (2022). Pustular psoriasis appearing induced by dupilumab therapy in a patient with atopic dermatitis. J Drugs Dermatol.

